# Influence of High-Temperature Substrate Preheating on Laser Cladding of Stellite 6 onto Inconel 718 Alloy

**DOI:** 10.3390/ma18081814

**Published:** 2025-04-15

**Authors:** Andrzej Gradzik, Karol Walczyk, Kamil Gancarczyk, Barbara Kościelniak, Mariusz Walczak, Natalia Gancarczyk, Jacek Nawrocki, Robert Albrecht

**Affiliations:** 1 Department of Materials Science, The Faculty of Mechanical Engineering and Aeronautics, Rzeszow University of Technology, 12 Powstancow Warszawy Ave., 35-959 Rzeszow, Polandkamilgancarczyk@prz.edu.pl (K.G.); b.koscielnia@prz.edu.pl (B.K.); jaceknaw@prz.edu.pl (J.N.); 2 Research and Development Laboratory for Aerospace Materials, Rzeszow University of Technology, Zwirki i Wigury Str., 35-036 Rzeszow, Poland; 3 Department of Materials Engineering, Faculty of Mechanical Engineering, Lublin University of Technology, 36 Nadbystrzycka Str., 20-618 Lublin, Poland; m.walczak@pollub.pl; 4 Doctoral School of the Rzeszow University of Technology, 12 Powstancow Warszawy Ave., 35-959 Rzeszów, Poland; d572@stud.prz.edu.pl; 5 Institute of Materials Engineering, University of Silesia in Katowice, 1a 75 Pulku Piechoty Str., 41-500 Chorzow, Poland; robert.albrecht@us.edu.pl

**Keywords:** laser cladding, Stellite 6, Inconel 718, preheating

## Abstract

Laser cladding is a modern surface treatment process used for the regeneration of damaged components and deposition of coatings for protection against corrosion and wear. Precise process control enables the production of claddings on small surfaces (<1 cm^2^). However, in some cases (e.g., cladding of turbine blades), there is a limited possibility of heat dissipation into the substrate material, which causes its rapid heating to several hundred degrees Celsius. This work’s objective is to determine the effect of the substrate temperature and laser cladding parameters of a Stellite 6 cobalt-based alloy on the Inconel 718 nickel-based alloy substrate on the geometry of a single cladding track, as well as its microstructure and hardness. Laser cladding with Stellite 6 powder was performed using an Yb:YAG TruDisk 1000 laser. The varied process parameters included the laser beam power density, cladding speed, and powder flow rate. The samples were preheated using a chamber furnace to a temperature ranging from 20 to 800 °C. The geometry of the single tracks produced by laser cladding and the substrate material dilution ratio were determined by measurements conducted on their cross-sections. Further microstructure investigations were performed by means of electron microscopy (SEM). Additionally, hardness measurements (HV0.3) were conducted on the cross-section of each cladded track. It was found that a higher substrate temperature causes melt pool widening and increases the melt depth, while the height of the single cladded track remains only slightly altered. These phenomena lead to the excessive dilution of the substrate material in the cladding (>35%) and result in a decrease in its hardness to the values characteristic of the Inconel 718 substrate (395 HV0.3).

## 1. Introduction

Laser cladding is a modern surface treatment process used for the regeneration of damaged components and the deposition of coatings for protection against corrosion and wear. Laser cladding and related methods are also referred to as Laser Metal Deposition (LMD), Direct Metal Deposition (DMD), Directed Energy Deposition (DED), and Laser Engineered Net-Shaping (LENS^®^). These processes are currently also often used as an additive manufacturing method for metal alloys [1,2,3,4,5].

In the laser cladding process, the energy of the laser beam is used to generate the heat required for melting the substrate and coating material, usually provided in the form of powder or wire. One pass of the laser cladding head creates a single track with a width in the range of 0.3 to about 8.0 mm [1,2,3,4,5,6,7]. Claddings of more complex geometry consist of multiple partially overlapping tracks. The overlap is usually up to 50% of the width of the single track. The cladding thickness is strongly dependent on the height of the single track. A single layer of coating usually has a thickness from 0.05 to 2.00 mm [1,2,3,5].

Once the solidification of the liquid metal melt pool is complete, the chemical composition of the created cladding may be significantly different from the chemical composition of the substrate and deposited material. The degree of material mixing is defined by the substrate dilution ratio into the cladded layer. It is dependent on the parameters of the cladding process: the power density and velocity of the laser beam (cladding speed) as well as the volume of melted coating material and the substrate. The dilution ratio can be directly evaluated by a comparison of the substrate and powder chemical compositions or evaluated using the metallographic method. The second method is based on the planimetric evaluation of the volume fraction of the substrate. Measurements are carried out on the cross-section of the single track or the coating [1,8,9,10]. To sum up, the critical parameters controlling the output of the laser cladding process are the beam power and size on the substrate surface, which determines the power density, cladding speed, and volume of the melted powder. The latter is regulated by the powder flow rate and absorbed energy. The process parameters affect the geometry of a single track. The most important geometrical features of the single track are the height, width, depth, cross-section area, and dilution ratio. These are the main factors deciding the final properties of the laser-cladded coating [1,2,8,9,10,11,12,13,14].

It should be noted that apart from laser cladding process parameters such as the beam power and diameter, cladding speed, and powder flow rate, other important aspects determine the achieved results. Among them are the physical properties of the substrate and the cladding material, substrate temperature, processing gases, and powder particle shape and size. Hardware considerations should also be taken into account due to the wide variety of laser radiation sources, machine tools, and laser cladding heads presently available on the market. Therefore, in order to improve the quality of empirically based mathematical models of the laser cladding process, it is necessary to constantly supplement the data presented in the literature with the results of research [1,2,8,9,10,11,12,13].

The laser cladding process has two unique features, the first of which is that it is a much more precise process control compared to conventional plasma and arc cladding methods such as TIG and MIG/MAG. The second one is its capability to produce precise cladding layers on small surfaces (<1 cm^2^). Such a process is often performed on the contact surfaces of shrouded gas and steam turbine blades, which are relatively thin-walled elements. There is a limited possibility of heat dissipation into the turbine blade. Therefore, there is the possibility of a significant change in the substrate temperature during the process, even by several hundred degrees Celsius in a short time. The cladding process therefore requires additional consideration of this phenomenon [2,5,15,16].

In the above-mentioned process, the substrate is the turbine blade material—usually a heat-resistant nickel-based alloy such as Inconel 713C, Inconel 738LC, Inconel 718, Mar M-200, or Mar M-247, with large equiaxed or columnar grains. The cladding layer is made of a Co-Cr-W-C alloy. It is usually one of the Stellite alloys such as Stellite 694 (CM64), Stellite 6, Stellite 21, and others. These alloys show excellent high-temperature wear and corrosion resistance, as well as resistance to cavitation. The production of these cladded layers is also intended to improve the fretting resistance [17,18,19,20,21,22].

Some studies have investigated the effect of preheating on the Stellite laser cladding process, primarily focusing on reducing the cracking susceptibility. The influence of the substrate temperature on the cladding geometry and dilution ratio, which ultimately determine the layer’s properties, has received less attention so far. Data on Stellite 6 and many Co-Cr-W-C alloys cladding are available in several publications, including, among others, an analysis of the pulsed cladding process. Sun et al. [23] investigated the effects of the pulse energy, pulse frequency, powder mass flow rate, and spot overlap on the cladding layer height, dilution ratio, and heat-affected zone (HAZ). Wu et al. [24] investigated the effect of preheating the substrate surface up to 300 °C for the laser cladding of Stellite 6+WC powder on 60Si2Mn steel. The authors showed that preheating improved the hardness and wear resistance of the Stellite 6+WC coating. The authors also proved that preheating impacts the geometric features of single tracks produced by the laser cladding process. Jendrzejewski et al. [25] conducted simulations of the temperature and stress fields during the laser cladding of Stellite SF6. They included preheating up to 500 °C as a factor. The results showed that stress reduction caused by preheating the substrate surface to 500 °C resulted in crack-free claddings. Zanzarin et al. [26] showed that during the laser cladding of Stellite 6+WC, preheating can increase carbide dissolution in the matrix due to the higher melt pool temperature. A reduction in residual stresses by using a substrate preheated to 200 and 300 °C has been demonstrated by Ya et al. in [27]. Fallah et al. [28] used localized preheating to overcome the cracking susceptibility in the laser cladding of high-carbon Stellite 1 alloy on AISI 4340 steel. The authors used a laser beam to locally preheat the surface prior to the cladding process. The actual substrate temperature was not measured in that particular work, though.

The aim of this work is to determine the effect of the substrate preheating and laser cladding parameters of the Stellite 6 cobalt alloy on the Inconel 718 nickel superalloy substrate on the geometry of a single track as well as its microstructure and hardness The input parameters included the laser beam power density, cladding speed, powder flow rate, and substrate temperature ranging from 20 to 800 °C.

## 2. Materials and Methods

Inconel 718 nickel-based alloy (Special Metals Corporation, New Hartford, NY, USA) was used as the substrate material (Table 1). Inconel 718 is a precipitation-hardened alloy known for its excellent corrosion resistance and high strength [29,30,31]. It is readily weldable by most arc and high-energy beam welding processes. Samples, 10 mm thick, were cut from 40 mm bars using a water-cooled band saw. The sample surfaces were then ground with Al_2_O_3_ FEPA P100 grit sandpaper (Struers ApS, Ballerup, Denmark). Prior to the laser cladding process, the samples were cleaned ultrasonically with acetone for 10 min and then dried with compressed air.

**Figure 1:**
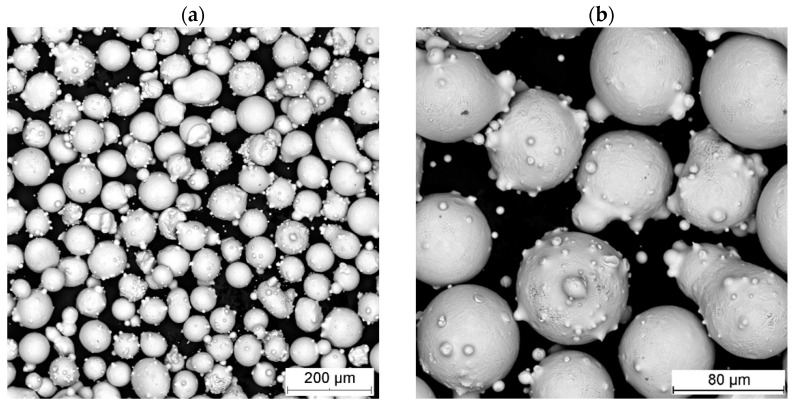
Morphology of Stellite 6 powder used in this research (**a**), satellites present on the particles surface (**b**).

Stellite 6 (Co-28Cr-4W-3Ni-3Fe-1.5Si-1C) cobalt alloy powder was used for laser cladding. The powder was acquired from Oerlikon Metco (Wohlen, Switzerland) as *Metcoclad 6*. The particle size of the powder was 44 ÷ 106 μm. Prior to the cladding process, the powder was dried in an oven at 100 °C for 60 min. The Stellite 6 powder was produced using a gas atomization process, and had a spherical particle shape (Figure 1).

**Figure 2:**
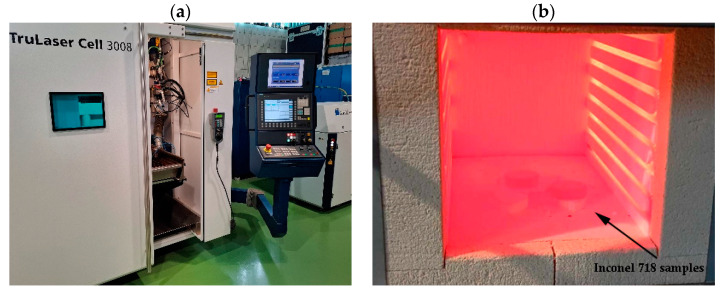
Laser equipment used in the research (**a**) and substrate samples placed in the furnace for the preheating process (**b**).

Laser cladding of the sample surfaces was performed using an Yb:YAG TRUMPF TruDisk 1000 laser with a maximum power of 1 kW and wave length *λ* = 1030 nm and a TruLaser Cell 3008 workstation (TRUMPF Laser GmbH + Co., Ditzigen, Germany) at the Research and Development Laboratory for Aerospace Materials at Rzeszow University of Technology (Figure 2a). A fiber optic cable with diameter of 200 μm and BEO D70 optics including a three beam nozzle cladding head was used in the process. The focal length of the lens was 220 mm, the working distance from the substrate was 12 mm, and the powder stream diameter at the workpiece was 2.5 mm. The powder feeder applied for cladding was GTV PF2/1 incorporated in the workstation system. Laser cladding processes were performed with the parameters used as listed in Table 2. Argon (>99.995% pure) was used as shielding gas (flow rate 15 dm^3^/min). Helium of the same purity degree was used as a powder carrier gas (flow rate 6 dm^3^/min). The applied combinations of laser cladding process parameters resulted in a total of 128 processes to be carried out in the research. Preheating of the samples was performed in a Carbolite chamber furnace (Carbolite Gero GmbH & Co. KG, Hope, UK). Samples were heated up to the desired preheat temperature plus 30 °C, then held for 20 min and transferred to the laser workstation (Figure 2b). The total time between taking the samples out of the furnace and starting the laser cladding process was maintained below 10 s.

**Figure 3:**
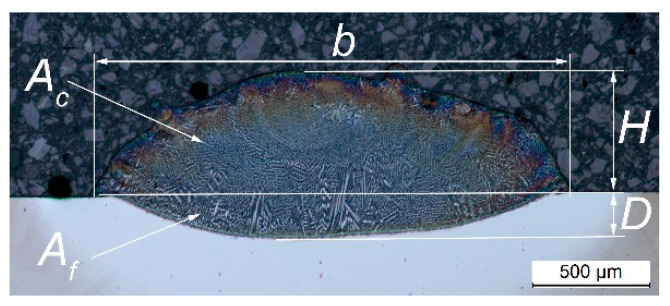
Geometric features of the laser-cladded track measured on the cross-section.

Geometric parameters and features of the laser-cladded Stellite 6 tracks were measured on their cross-sections. The following parameters were analyzed (Figure 3): width of the track—*b*; height of the track (above the substrate surface)—*H*; depth of fusion—*D*; cross-section area surface of the track (above the substrate surface)—*A_c_*; and cross-section area of the melted substrate—*A_f_*.

The dilution ratio was calculated using the formula including *A_c_* and *A_f_* measured on the cross-section of the single track (1). It was assumed that stereologically, the dilution corresponds to the volume fraction (*V_v_*) of the substrate in the clad.

(1) $$V_{v}=\frac{A_{f}}{A_{C}+A_{f}}$$

The surface of the cladded tracks was observed using a stereoscopic microscope OPTA-TECH X2000 (OPTA-TECH, Warszawa, Poland). Microstructure examination of samples was performed with a Leica DMI 3000M (Leica Microsystems, Wetzlar, Germany) metallographic microscope. Metallographic sample preparation included sectioning with a water-cooled abrasive cut-off machine, wet grinding with diamond disks (80 ÷ 2000 grit), polishing with 3 μm, and then 1 μm diamond suspension. Etching was performed electrolytically using 150 cm^3^ HCl + 50 cm^3^ C_3_H_6_O_3_ + 3g C_2_H_2_O_4_·2H_2_O, U = 3V, t = 2 ÷ 4 s. Scanning electron microscopy investigations included microstructure examinations using Phenom XL (Thermo Fischer Scientific, Waltham, MA, USA).

The hardness measurements of the laser-cladded samples were performed on the cross-section, using the Innovatest Nexus 4303 (Innovatest, Maastricht, The Netherlands) hardness tester. Measurements were carried out with a Vickers indenter and 2.94 N (HV0.3) load. Before the surface hardness measurements, the samples were polished with a diamond suspension. The average of 4 hardness indentations was determined for each sample.

## 3. Results and Discussion

### 3.1. Surface of the Cladded Samples

**Figure 4:**
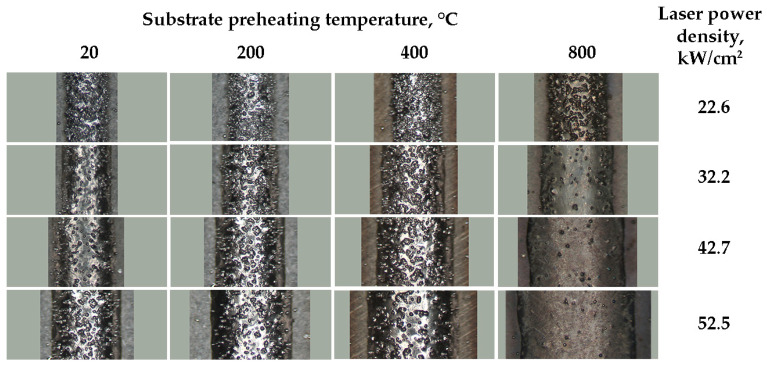
The surface of the Stellite 6 laser-cladded tracks at power density 22.6 ÷ 52.5 kW/cm^2^ and preheat temperature 20 ÷ 800 °C. Powder flow rate 4.0 g/min, cladding speed 300 mm/min. Picture background (grey) dimensions 1.9 × 4.5 mm.

**Figure 5:**
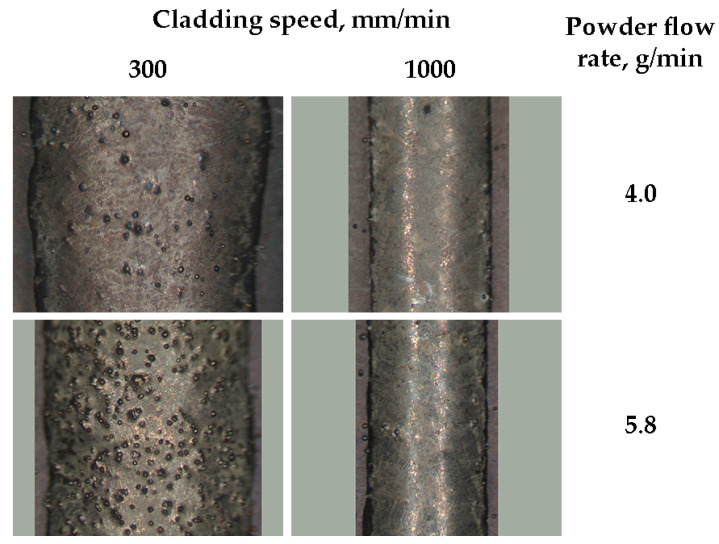
Influence of cladding speed and powder flow rate on the partially melted powder residues on the surface of the cladded track. Laser beam power density 52.5 kW/cm^2^ and substrate temperature 800 °C. Picture background (grey) dimensions 4.0 × 3.1 mm.

The Stellite 6 laser-cladded tracks were observed using a stereoscopic microscope, maintaining constant magnification. The surface analysis showed the presence of partially melted powder particles. The observations indicate that the substrate preheating temperature, powder flow rate, cladding speed, and power density influence the number of these particles. The number of partially melted powder particles present at the surface notably decreased as the substrate temperature rose from 20 to 800 °C, while the other process parameters remained constant (Figure 4). However, an increase in the powder flow rate led to a corresponding rise in the number of partially melted powder residues on the surface. This suggests that the powder flow rate of 5.8 g/min, as utilized in the experiments, exceeds the optimal level under the defined laser cladding parameters. Consequently, at this flow rate, a higher laser beam power is necessary, especially for a laser cladding speed of 300 mm/min. At its higher values, the reduced volume of the delivered powder minimizes the remaining unmelted and partially melted particles on the cladded track surface (Figure 5). At each laser power density (22.6 ÷ 52.5 kW/cm^2^), there is a minor increase in powder particle residues on the track surface between the substrate preheating temperature from 20 to 400 °C.

This phenomenon is presumably associated with the longer solidification time of the melt pool and thus a prolonged period of sufficiently high cladding surface temperature, which enables powder particle adhesion. Under these conditions, the melt pool is still much narrower than the Stellite 6 powder stream at the sample surface. For substrate preheating to 800 °C, the melt pool width increases to values similar to the powder stream diameter (2.5 mm), resulting in a reduction in powder residues on the cladding surface.

### 3.2. Geometry of the Cladded Tracks

The microscopic examination of the laser-cladded track cross-section geometry showed correct substrate melting in 97% of the samples. Incomplete fusion was observed at a power density of 22.6 kW/cm^2^, a powder flow rate of 5.8 g/min, and a cladding speed ranging from 300 to 1000 mm/min (Table 3). However, preheating the substrate to 200 ÷ 800 °C enabled the achievement of complete fusion for these laser cladding process parameters. As this issue was not present at a powder flow rate of 4.0 g/min, it is likely attributed to increased laser radiation absorption by the powder particles, resulting in insufficient energy transfer to the substrate for melting and achieving the desired liquid metal pool width and depth. The issue of incomplete substrate fusion was resolved by increasing the laser beam power density. In the current study, it was noted that raising the power density to 32.2 kW/cm^2^ was sufficient to prevent the lack of fusion of the substrate, regardless of other laser cladding parameter values.

**Figure 6:**
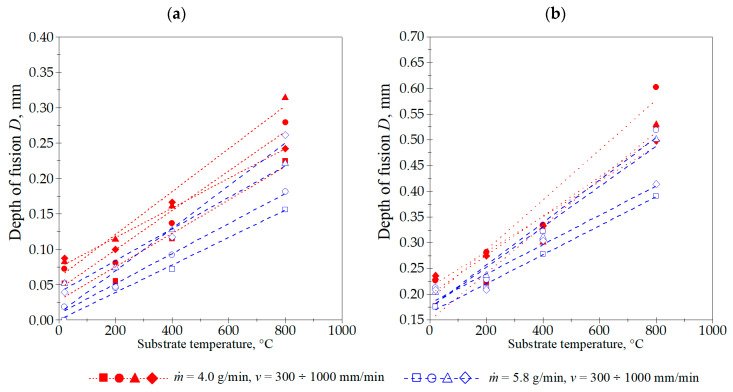
Influence of cladding speed *v*, powder flow rate *ṁ*, and substrate preheating temperature on the depth of fusion *D*, for laser beam power density: (**a**) 22.6 kW/cm^2^, (**b**) 52.5 kW/cm^2^.

**Figure 7:**
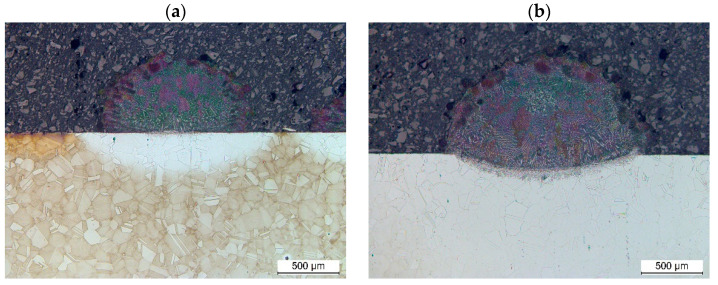
Effect of substrate temperature on susceptibility to the lack of fusion defect occurrence during laser cladding. Process parameters: laser power density 22.6 kW/cm^2^, powder flow rate 5.8 g/min, cladding speed 300 mm/min, and substrate temperatures 20 °C (**a**) and 800 °C (**b**).

**Figure 8:**
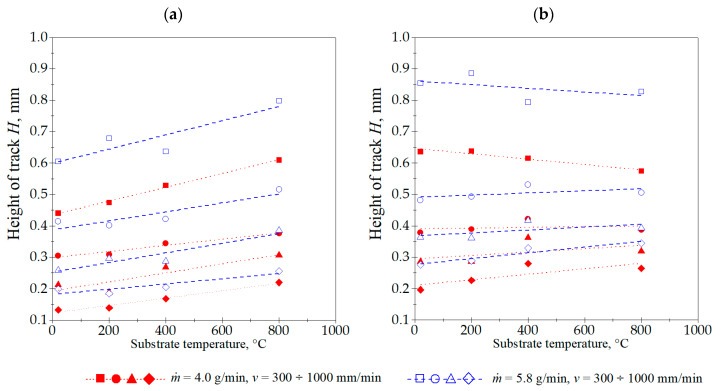
Influence of cladding speed *v*, powder flow rate *ṁ*, and substrate preheating temperature on the height of the single track *H*, with laser beam power densities of (**a**) 22.6 kW/cm^2^ and (**b**) 52.5 kW/cm^2^.

**Figure 9:**
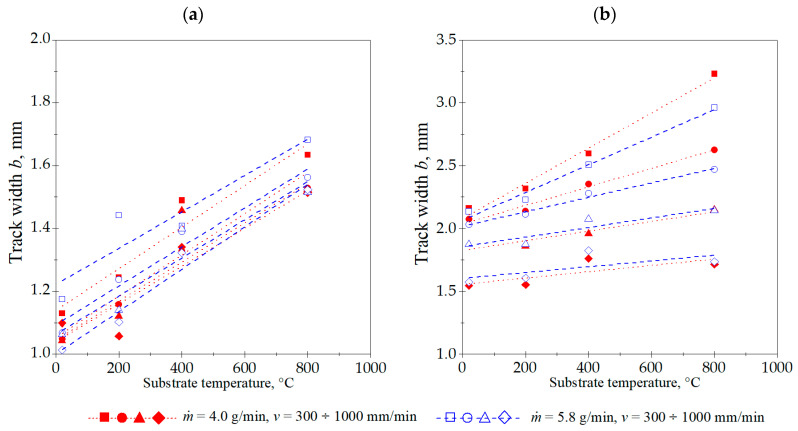
Influence of cladding speed *v*, powder flow rate *ṁ*, and substrate preheating temperature on the width of the single track *b*, with laser beam power densities (**a**) 22.6 kW/cm^2^ and (**b**) 52.5 kW/cm^2^.

**Figure 10:**
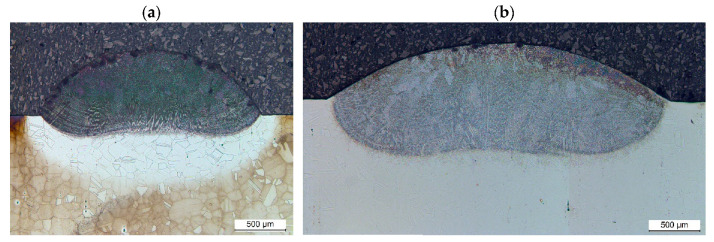
Effect of substrate temperature on height and width of single cladded track. Process parameters: laser power density 52.5 kW/cm^2^, powder flow rate 4.0 g/min, cladding speed 300 mm/min, and substrate temperatures 20 °C (**a**) and 800 °C (**b**).

The laser cladding process parameters utilized in these experiments produced cladding layers with the maximum and minimum dimensions and characteristics, as detailed and summarized in Table 3. The cross-sectional geometric measurements of the cladding layers revealed distinct correlations between these dimensions and the laser cladding process parameters (Figure 6, Figure 7, Figure 8, Figure 9 and Figure 10).

The Stellite 6 cladded track geometry measurements indicate that raising the laser beam power density correlates with an increased depth of the melted substrate layer. The track height also increases, notably between 22.6 and 42.7 kW/cm^2^. However, a further increase to 52.5 kW/cm^2^ yields no significant change in the cladded track height. The laser power density also determines the liquid metal pool width and, consequently, the final track width. This width increases consistently with the power density. The cladding speed v also has an impact on the track width, although a substantial reduction in its value is only observed at 1000 mm/min, provided that the substrate temperature and other laser cladding process parameters remain constant. Similar trends were observed for parameters *A_c_* and *A_f_*, which are partially related to the basic dimensions of the single track: *b*, *H*, and *D*.

The studies showed a significant increase in all dimensions and the cross-sectional area of the single tracks produced by laser cladding with the preheated substrate material (Inconel 718 alloy). Increasing the substrate temperature allowed for the successful melting of the substrate at a power density of 22.6 kW/cm^2^, even with a powder flow rate of 5.8 g/min.

Most importantly, the substrate temperature exhibited a combined effect on both the melt depth and width of the track. Both parameters increased significantly with the rise in the initial substrate temperature. However, the track height not only did not increase significantly when the substrate temperature was raised, but, in some cases, was reduced. This effect was particularly noticeable at a preheating temperature of 800 °C. For example, laser cladding at *v* = 300 mm/min, *ṁ* = 4.0 g/min, and power density of 52.5 kW/cm^2^, at substrate temperature of 20 °C produced a single track with the following dimensions: *b* = 2.16 mm, *D* = 0.18 mm, and *H* = 0.64 mm. Preheating the substrate to 800 °C altered these parameters to *b* = 3.23 mm, *D* = 0.52 mm, and *H* = 0.57 mm. In this case, there was an increase in the track width and melt depth by 49 and 196%, respectively, while the depth of fusion dropped by 10%. This suggests that excessive substrate melting occurring at higher substrate temperatures would lead to an increased dilution ratio (*V_v_*), defined by the volume fraction of substrate in the clad. Further investigations indicated that this resulted in a decrease in the single track hardness.

### 3.3. Microstructure of the Cladded Tracks

**Figure 11:**
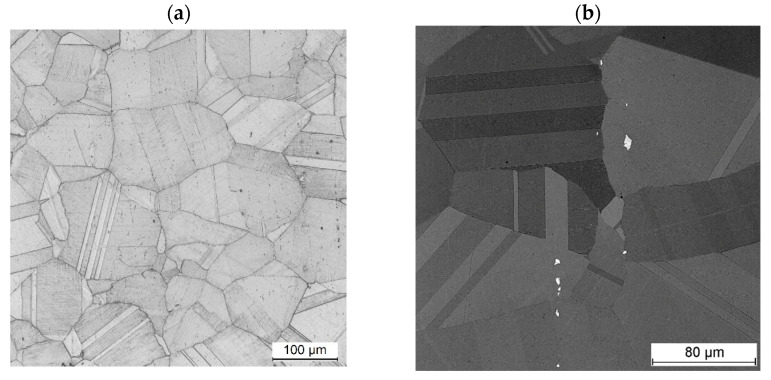
Microstructure of substrate material Inconel 718 showing (**a**) equiaxed grains and (**b**) precipitates present in the microstructure (SEM/BSE).

The substrate material, nickel alloy Inconel 718, used in this research, had an isotropic microstructure composed of equiaxed grains. The twin boundaries were widely present within the grains. It was found that the carbonitride precipitations, typical for this alloy, form a banded structure along the axis of the rod from which the substrate samples were cut [28,29,30,31]. The δ-phase precipitations were not detected at the grain boundaries (Figure 11).

**Figure 12:**
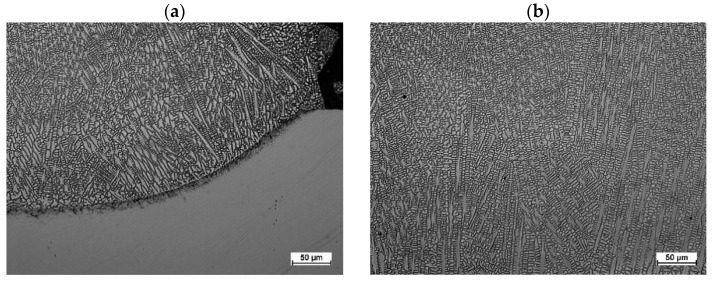
Microstructure of Stellite 6-cladded single track on the Inconel 718 substrate showing fusion boundary (**a**) and dendritic microstructure (**b**).

**Figure 13:**
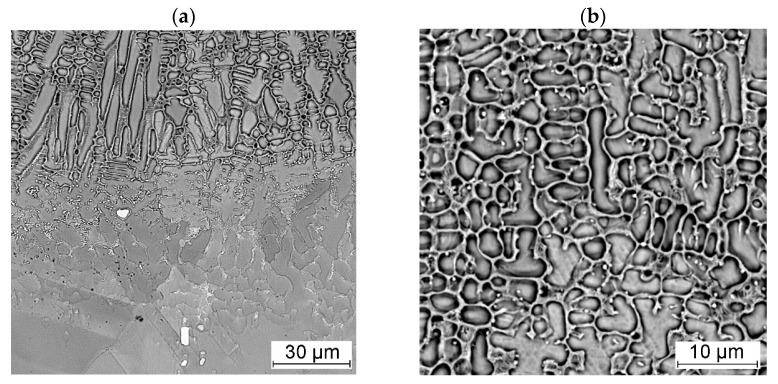
Microstructure of Stellite 6-cladded single track on the Inconel 718 substrate showing (**a**) growth of dendrites at the fusion boundary and (**b**) dendritic microstructure with precipitates in the interdendritic areas (SEM/BSE).

The Stellite 6-cladded track on the Inconel 718 superalloy substrate had a dendritic structure (Figure 12 and Figure 13). The dendritic growth was initiated at the fusion boundary and proceeded in the direction of the moving heat source—the laser beam. The rapid melting and crystallization of the liquid metal during laser cladding limited the fragmentation of the dendrites and the growth of secondary dendritic arms.

**Figure 14:**
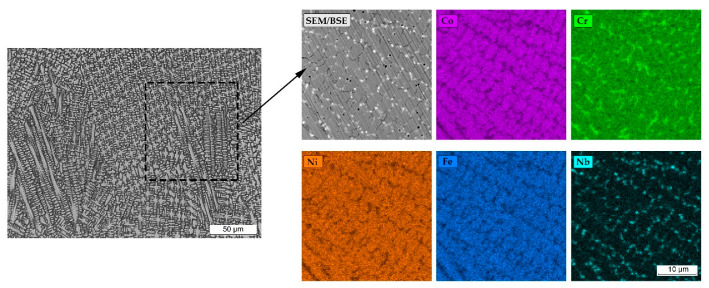
Microstructure of Stellite 6-cladded single track on the Inconel 718 substrate and EDS mapping of the element content in the dendrites and interdendritic areas.

An increased concentration of chromium, molybdenum, niobium, and titanium was observed in the interdendritic areas of the cladded tracks (Figure 14). It should be assumed that chromium-rich carbide precipitates are formed in the interdendritic areas during the crystallization of the alloy. Based on the analysis of data in the literature, it was determined that the Stellite 6 cobalt alloy is characterized by a tendency to form carbide precipitates of the following types: M_23_C_6_; M_7_C_3_; and M_6_C [32,33]. No effect of the initial substrate temperature on the changes in the dendritic crystallization front at the fusion boundary (e.g., to flat or cellular) was observed. Niobium-rich precipitate formation was observed at the partially melted zone (Figure 13a).

**Figure 15:**
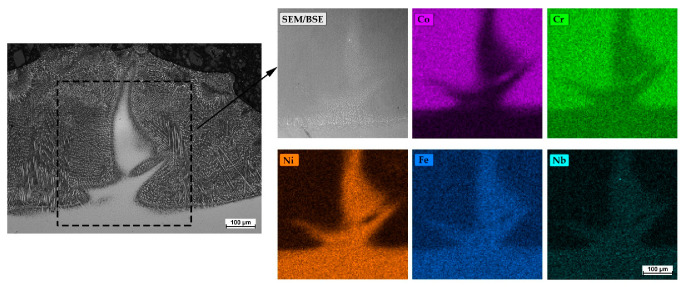
Microstructure of Stellite 6 single track laser-cladded on the Inconel 718 substrate and EDS mapping of the element concentration within chemical composition inhomogeneity area in the central part.

It was observed that the single tracks produced at a cladding speed of 300 mm/min exhibited chemical composition inhomogeneity. This phenomenon was observed in the central part of the cladded track (Figure 15) and was related to the convection of liquid metal in the melt pool. The analysis of the chemical composition showed significantly increased contents of Ni, Fe, and Nb in this region. The effect of incomplete cladding homogenization was not observed when the substrate was preheated to 800 °C.

### 3.4. Effect of Dilution Ratio on Hardness of Cladded Single Tracks

The Stellite 6 laser-cladded single tracks exhibit various dilution ratios (*V_v_*) depending on process parameters such as the power density, cladding speed, and substrate temperature. Increasing the power density is associated with a rise in the thermal energy generated in the process. This, in turn, leads to more intensive melting of the substrate material. An increase in the cladding speed favors a higher dilution ratio by reducing the volume of powder supplied to the pool of liquid metal per unit of time. In turn, as the temperature of the substrate material rises, the energy required to heat the substrate to the liquidus temperature decreases, which leads to excessive substrate melting and the widening of the melt pool. With the increase in these parameters, the processes of diffusion in the liquid state occurring during laser cladding are intensified. Therefore, significant changes in the alloying element concentration in the cladded track can be observed.

**Figure 16:**
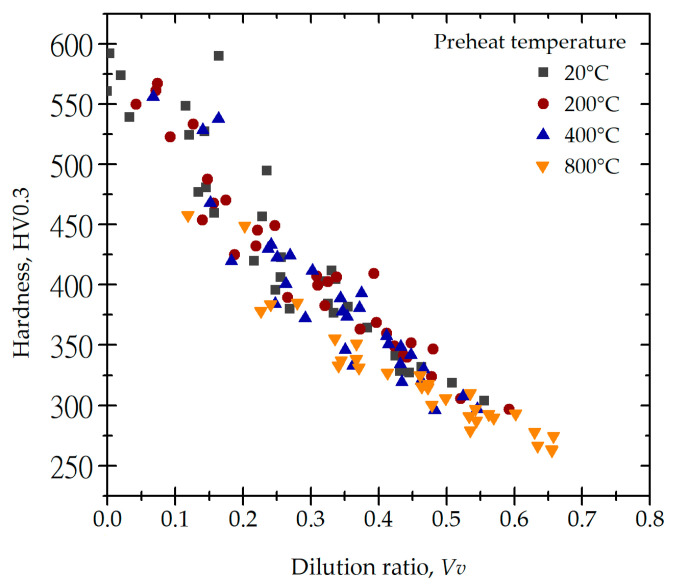
Influence of dilution ratio on the Stellite 6 laser-cladded track hardness for substrate temperatures ranging from 20 to 800 °C.

The Stellite 6 laser-cladded tracks on the Inconel 718 alloy substrate achieve a much higher hardness than that of the substrate material with certain process parameters. The hardness of the substrate material was 395 ± 17 HV0.3 (T = 20 °C). However, after preheating to T = 800 °C, the hardness of the substrate decreased to 300 ± 12 HV0.3. The current studies have shown that the hardness of the produced Stellite 6-cladded track depends, to a great extent, on the dilution (*V_v_*). The obtained experimental data indicate the synergy effect of the laser cladding process parameters, leading to the obtainment of a specific degree of mixing of the substrate and melted Stellite 6 powder (Table 4, Table 5, Table 6, Table 7 and Table 8, Figure 16).

The highest hardness, 592 HV0.3, was obtained for single cladded tracks produced under the following process conditions: substrate material temperature *T* = 20 °C, power density = 22.6 kW/cm^2^, and cladding speed 300 and 500 mm/min. Nevertheless, these cladding parameters simultaneously led to the lack of fusion at the substrate–cladding interface. In this case, *V_v_* ≈ 0, and the obtained hardness value corresponds only to the hardness of the melted Stellite 6 powder. The lowest hardness (approx. 275 HV0.3) of the cladded track was obtained for the following process conditions: substrate temperature *T* = 800 °C, laser power density = 52.5 kW/cm^2^, and cladding speed 700 and 1000 mm/min. The dilution ratio at these conditions rose to *V_v_* = 0.65.

The measurements of the geometric dimensions and hardness of the Stellite 6-cladded single track, as well as the microstructure observations, lead to the reasoning that numerous cladding process-related variables and phenomena are affecting the final hardness. The synergistic effect of the laser beam power density and substrate preheating at temperatures of 200, 400, and 800 °C promotes the expansion of the molten pool and induces excessive melting of the substrate material. This, in turn, causes a significant increase in the dilution ratio (*V_v_*) (Figure 16). The Stellite 6 cobalt alloy exhibits a carbon concentration of approximately 1.0 wt.%, whereas the Inconel 718 substrate contains a relatively low carbon concentration of 0.013 wt.%. The diffusion of constituent elements within the liquid metal pool results in a net reduction in the carbon content in the cladding layer. The available data in the literature indicate the crucial role of carbides in the hardness of the cladded layer. For the Stellite 6 alloy, these precipitates are predominantly chromium-rich M_7_C_3_ and M_23_C_6_ carbides [32,33,34,35]. The observed decrease in their volume fraction within the cladding layer, attributable to elevated dilution levels, correlates with a reduction in the final hardness [34]. A low dilution ratio is also advantageous due to the possibility of microstructure refinement associated with rapid cooling [34]. The analysis of the acquired data reveals that a critical threshold for a substantial decrease in hardness is surpassed beyond *V_v_* = 0.15 (15% of the substrate material in the cladding). At this value, the hardness decreases to about 450 HV0.3, which is also ca. 76% of the highest hardness obtained in this study: 592 HV0.3. This dilution ratio is also one of the lowest obtained for the preheating temperature of 800 °C, possible to achieve only at a laser power density of 22.6 kW/cm^2^. Based on these results, it is therefore suggested to consider in-process modulation of the laser cladding process parameters by reducing the power density as the substrate temperature increases in order to maintain a constant dilution ratio.

## 4. Summary and Conclusions

This research focused on determining the effect of a high substrate temperature on the dimensions of the single track produced in the laser cladding process of Stellite 6 onto Inconel 718 alloy. The acquired and analyzed data are of importance from the point of view of cladding processes carried out under conditions of a small working surface (<1 cm^2^) and limited possibilities of heat dissipation into the substrate. An example of such a process is Stellite cladding of Z-notched turbine blades, where process control is required to obtain a high hardness of the protective cladded layer. The following conclusions can be drawn from this work:

1. The temperature of the substrate material significantly affects the dimensions of a single track produced by laser cladding Stellite 6 powder onto the nickel alloy Inconel 718. A higher substrate temperature promotes a more intensive melting process of the substrate material. The width of the single track and the depth of substrate melting increase while the height of the cladded track remains at a similar level. These phenomena lead to a higher dilution ratio of the substrate material.

2. Laser cladding process parameters: the cladding speed, power density, and powder flow rate affect the dimensions of a single track to a varying extent. The laser beam power density determines the width of the cladding and the depth of the melted substrate layer. On the other hand, the cladding speed and powder flow rate have the greatest effect on the height of the cladded track.

3. The applied laser beam power density of 22.6 kW/cm^2^ is insufficient to obtain the correct melting of the substrate material in the cladding process with Stellite 6 powder on the Inconel 718 superalloy substrate, while the remaining process parameters are applied as in the conducted experiment. At the same time, increasing the temperature of the substrate material by preheating to >200 °C prevents the formation of this defect.

4. The Stellite 6-cladded tracks have a dendritic structure with carbide precipitations developing in the interdendritic areas. It was determined that under certain conditions of the cladding process, there is the possibility of Ni-, Fe-, and Nb-enriched zone formation in the central part of the track. This is due to the convection-related transfer of alloying elements from the substrate to the clad.

5. An increase in the substrate material dilution ratio (*V_v_*) in the cladded track significantly reduces its hardness. At dilution ratio values higher than 0.35, the cladding hardness decreases below the hardness of the substrate.

The research results acquired in this work enable their application in laser cladding processes in industrial engineering. This includes initial guidelines for real-time process control, which would involve power density corrections based on substrate temperature measurements to maintain a similar dilution ratio and constant cladding hardness. Further work on the subject of the substrate temperature’s influence on the Stellite 6 cladding process will incorporate modeling using statistical techniques such as multiple regression, as well as increasingly popular machine learning methods. This approach will allow for a deep understanding of the studied correlations and the development of accurate predictive models.

## Figures and Tables

**Table 1:** Chemical composition of the Inconel 718 alloy used in research.

Element, wt.%									
C	Fe	Cr	Mo	Nb	Si	Ti	Al	Co	Ni
0.013	17.84	17.64	2.95	5.00	0.05	0.99	0.48	0.31	Bal.

**Table 2:** Laser cladding process parameters.

Laser Cladding Parameter	Value
Laser beam power, W	300; 428; 566; 698
Laser beam diameter *d*_0_, mm	1.30
Laser power density, kW/cm^2^	22.6; 32.2; 42.7; 52.5
Cladding speed *v*, mm/min	300, 500, 700, 1000
Powder flow rate *ṁ*, g/min	4.0; 5.8
Substrate temperature *T*, °C	20; 200; 400; 800

**Table 3:** Summary of geometric parameters and features of single tracks produced by laser cladding of Stellite 6 on the Inconel 718 substrate.

Geometric Parameters and Features	Powder Flow Rate			
	4.0 g/min		5.8 g/min	
	Min.	Max.	Min.	Max.
Track width—*b*, mm	1.04	3.23	1.01	2.96
Track height—*H*, mm	0.13	0.69	0.19	0.89
Depth of fusion—*D*, mm	0.05	0.60	0.00	0.52
Cross-section—*A_c_*, mm^2^	0.11	1.26	0.13	1.76
Cross-section of melted substrate layer—*A_f_*, mm^2^	<0.01	1.50	0.00	1.05

**Table 4:** Dilution ratio (*V_v_*) range occurring during laser cladding of Stellite 6 onto Inconel 718 substrate using parameters applied in this research.

Substrate Preheating Temperature *T*, °C	Dilution Ratio (*V_v_*)—For Respective Powder Flow Rates			
	4.0 g/min		5.8 g/min	
	Min.	Max.	Min.	Max.
20	<0.01	0.56	0.00	0.46
200	0.07	0.59	0.04	0.48
400	0.14	0.54	0.07	0.46
800	0.23	0.65	0.11	0.60

**Table 5:** Hardness (HV0.3) and corresponding dilution (*V_v_*) in relation to substrate preheating temperature and cladding speed for: laser power density 22.6 kW/cm^2^ and powder flow rate 4.0 g/min.

*v*, mm/min	Substrate Preheating Temperature, °C							
	20		200		400		800	
	HV0.3	*V_v_*	HV0.3	*V_v_*	HV0.3	*V_v_*	HV0.3	*V_v_*
300	592	<0.01	561	0.07	528	0.14	378	0.23
500	590	0.16	470	0.17	430	0.24	331	0.37
700	495	0.24	402	0.33	393	0.37	315	0.47
1000	403	0.32	409	0.39	389	0.48	291	0.53

**Table 6:** Hardness (HV0.3) and corresponding dilution (*V_v_*) in relation to substrate preheating temperature and cladding speed with laser power density 52.5 kW/cm^2^ and powder flow rate 4.0 g/min.

*v*, mm/min	Substrate Preheating Temperature, °C							
	20		200		400		800	
	HV0.3	*V_v_*	HV0.3	*V_v_*	HV0.3	*V_v_*	HV0.3	*V_v_*
300	396	0.25	389	0.27	389	0.34	287	0.54
500	341	0.42	340	0.44	319	0.43	264	0.65
700	319	0.51	305	0.52	296	0.48	263	0.66
1000	303	0.56	297	0.59	297	0.55	266	0.63

**Table 7:** Hardness (HV0.3) and corresponding dilution (*V_v_*) in relation to substrate preheating temperature and cladding speed with laser power density 22.6 kW/cm^2^ and powder flow rate 5.8 g/min.

*v*, mm/min	Substrate Preheating Temperature, °C							
	20		200		400		800	
	HV0.3	*V_v_*	HV0.3	*V_v_*	HV0.3	*V_v_*	HV0.3	*V_v_*
300	560	0.00	550	0.04	556	0.07	458	0.12
500	574	0.02	567	0.07	538	0.16	449	0.20
700	524	0.12	533	0.13	433	0.24	355	0.34
1000	527	0.14	487	0.15	412	0.30	355	0.50

**Table 8:** Hardness (HV0.3) and corresponding dilution (*V_v_*) in relation to substrate preheating temperature and cladding speed with laser power density 52.5 kW/cm^2^ and powder flow rate 5.8 g/min.

*v*, mm/min	Substrate Preheating Temperature, °C							
	20		200		400		800	
	HV0.3	*V_v_*	HV0.3	*V_v_*	HV0.3	*V_v_*	HV0.3	*V_v_*
300	459	0.16	425	0.19	424	0.27	351	0.37
500	404	0.34	406	0.34	351	0.41	297	0.54
700	385	0.41	341	0.44	348	0.43	293	0.60
1000	332	0.46	347	0.48	320	0.46	293	0.56

## Data Availability

The original contributions presented in this study are included in the article. Further inquiries can be directed to the corresponding author.
